# Precise protein quantification based on peptide quantification using iTRAQ™

**DOI:** 10.1186/1471-2105-8-214

**Published:** 2007-06-21

**Authors:** Andreas M Boehm, Stephanie Pütz, Daniela Altenhöfer, Albert Sickmann, Michael Falk

**Affiliations:** 1 Rudolf Virchow Center, DFG Research Center for Experimental Biomedicine, University of Wurzburg, (Protein Mass Spectrometry and Functional Proteomics), Wurzburg, D-97078, Germany; 2 Institute of Mathematics, University of Wuerzburg, Am Hubland, D-97074 Wuerzburg, Germany

## Abstract

**Background:**

Mass spectrometry based quantification of peptides can be performed using the iTRAQ™ reagent in conjunction with mass spectrometry. This technology yields information about the relative abundance of single peptides. A method for the calculation of reliable quantification information is required in order to obtain biologically relevant data at the protein expression level.

**Results:**

A method comprising sound error estimation and statistical methods is presented that allows precise abundance analysis plus error calculation at the peptide as well as at the protein level. This yields the relevant information that is required for quantitative proteomics. Comparing the performance of our method named Quant with existing approaches the error estimation is reliable and offers information for precise bioinformatic models. Quant is shown to generate results that are consistent with those produced by ProQuant™, thus validating both systems. Moreover, the results are consistent with that of Mascot™ 2.2. The MATLAB^® ^scripts of Quant are freely available via  and , each under the GNU Lesser General Public License.

**Conclusion:**

The software Quant demonstrates improvements in protein quantification using iTRAQ™. Precise quantification data can be obtained at the protein level when using error propagation and adequate visualization. Quant integrates both and additionally provides the possibility to obtain more reliable results by calculation of wise quality measures. Peak area integration has been replaced by sum of intensities, yielding more reliable quantification results. Additionally, Quant allows the combination of quantitative information obtained by iTRAQ™ with peptide and protein identifications from popular tandem MS identification tools. Hence Quant is a useful tool for the proteomics community and may help improving analysis of proteomic experimental data. In addition, we have shown that a lognormal distribution fits the data of mass spectrometry based relative peptide quantification.

## Background

Mass spectrometry is a common technique employed for protein identification in proteomics. In tandem mass spectrometry, proteins are identified by matching the measured fragment ion spectra of peptides with theoretical spectra calculated from known DNA or protein sequences [1], for example the NCBI sequence database [2] or Swiss-Prot [3].

Instead of studying a single protein in detail as done in former days of protein sciences, the analysis of all proteins of a cell – the proteome – became important [4]. The proteome comprises all the proteins present in an organism, tissue or cell at a particular time. In contrast to the genome, the proteome is not static but highly dynamic.

To understand the biological and biochemical processes in a cell or an organism, for example responses to different environmental influences or the difference between healthy and diseased tissue, analysis of all differences at genomic or proteomic level needs to be performed. The protein abundance changes over time are needed for understanding cellular processes [5].

Differences in protein expression are not accessible at genomic level but often are accessible at the proteome level [6]. Some proteins are up- or down-regulated in the different stages of a cell. Therefore, quantitative information of the expressed proteins is needed and constitutes a key-step to fully understand functions of organelles, cells, organisms as well as processes of diseases. Furthermore, the quantitative information of the protein expression can be used for bioinformatic modelling of cellular processes such as pathways, cell maturing and metabolisms [7].

**Figure 1:**
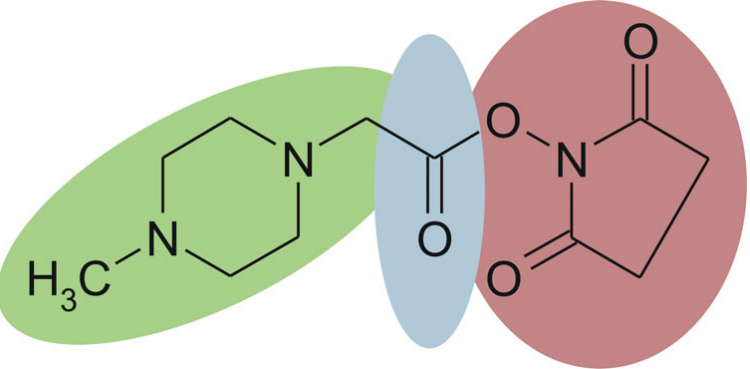
Chemical structure of the iTRAQ™ reagent. The label is composed of a peptide reactive group (red, NHS ester) and an isobaric tag of 145 Da, which consists of a balancer group (blue, carbonyl group) and a reporter group (green, N-methylpiperazine). The four available tags of identical overall mass vary in their stable isotope compositions such that the reporter group has a mass of 114–117 Da and the balancer of 28–31 Da. The fragmentation site between the balancer and the reporter group is responsible for the generation of the reporter ions in the region of 114–117 m/z.

The advantages of mass spectrometry-based peptide quantification are precision, sensitivity, throughput and convenient automation [8,9]. During the last decade, several techniques have been established [10], e.g. the isobaric tag for relative and absolute quantitation (iTRAQ™) that is currently the only technique capable of multiplexing up to four different samples for relative quantification. Four chemically identical iTRAQ™ reagents are available, named 114, 115, 116, 117, which have the same overall mass. Each label is composed of a peptide reactive group (NHS ester) and an isobaric tag of 145 Da that consists of a balancer group (carbonyl) and a reporter group (based on N-methylpiperazine) [11], as shown in figure 1. Between the balancer and the reporter group is a fragmentation site. The peptide reactive group attaches specifically to free primary amino groups – N-termini and ε-amino groups of lysine residues. Side reactions on tyrosine have been also reported [11]. No labelling occurs if the primary amino groups are modified, for example N-terminal glutamine or glutamic acid could form a ring (pyroglutamic acid) or an acetylation may occur. Therefore by using iTRAQ™, peptides within the sample are labelled that possess at least one free primary amino group.

In fragment ion spectra of iTRAQ™ labelled peptides, additional peaks appear in the m/z range of 114 to 117, originating from the singly charged reporter group fragment of each iTRAQ™ label. Peptide quantification can be performed by interpretation of these peaks. In order to allow for judging the results calculated from the reporter peaks, a reliable quality measure is needed [12] not only at the peptide level.

The development of precise and transparent methods for analysis of proteomic data is one of the crucial challenges in protein sciences [8]. A software for data evaluation support is needed for quantification, because Proteomics yields huge amounts of data [13]. These computer programs must be capable of providing results at the protein level. Some software already is available for analyzing iTRAQ™ data, such as i-Tracker [14], MassTRAQ [15], ProQuant™ (Applied Biosystems (ABI), Darmstadt, Germany), ProteinPilot™ (ABI) or Mascot™ 2.2 (Matrix Science, London, UK). Some of these are not freely available, such as ProQuant™, ProteinPilot™ and Mascot™. MassTRAQ and i-Tracker only provide data at the peptide level. These tools have in common that they are not capable of calculating reliable quantification information at the protein level or do not provide precise error estimation or a reliable quality measure. Some of them assume a mismatching and inappropriate distribution for their peptide and signal statistics. We thus decided to develop our tool named Quant for quantification at peptide level as well as at protein level. We focus on the protein level, as only this allows meaningful interpretations of the experimental data including a reliable transfer into bioinformatic modelling. Moreover, this software is freely available.

## Methods

### Experiments

The functionality of Quant has been proven by application to a standard protein mix provided by Applied Biosystems within the iTRAQ™ kit.

### Sample preparation

A six-protein mix delivered with the iTRAQ™ kit was used for the analysis. The protein mix consisted of bovine serum albumin (Accession Number P02769), β-galactosidase (P00722), α-lactalbumin (P00711), β-lactoglobulin (P02754), lysozyme (P00698), apo-transferrin (P02787).

The proteins were dissolved according to the iTRAQ™ reagent protocol [16] in 100 mM triethylammonium bicarbonate buffer at pH 8.5. The cysteine residues were blocked and alkylated with MMTS as described in the iTRAQ™ protocol and the proteins were digested overnight using trypsin. The obtained peptides were labelled with the iTRAQ™ reagent in 70% ethanol.

The sample was divided in two sections, whereby one half was labelled with the iTRAQ™ reagent 114 and the other with 117. These differently labelled samples were mixed 1:1 and 1:3. The samples were separated by using multidimensional liquid chromatography. In the first dimension, the mixture was separated by strong cation exchange  chromatography (PL-SCX; 2.1-mm inner diameter (ID), 150-mm length, 1000-Å  pore size, 8-μm particle size, Polymer Laboratories, Darmstadt, Germany) using  a linear binary gradient (solvent A: 50 mM KH_2_PO_4_, pH 3.5; solvent B: 50 mM  KH_2_PO_4_, 0.25 M NaCl, 25% ACN, pH 3.5). The separation of the peptides was performed with a gradient of 2% per minute increasing amount of solvent B. SCX fractions were taken every minute and the organic solvent was removed under vacuum, furthermore the fractions were separated in a second dimension and analyzed using nano LC-MS/MS.

The nano MS/MS analysis was conducted with a Qstar XL (ABI). Samples were preconcentrated using a C18 Pep-Map trapping column (300 μm ID, 1 mm length, 100 Å pore size, 5 μm particle size; Dionex, Idstein, Germany) and afterwards separated on a C18 PepMap main column (75 μm ID, 150 mm length, 100 Å pore size, 3 μm particle size; Dionex) using a linear binary gradient (solvent A: 0.1% FA; solvent B: 0.1% FA, 84% ACN). Full MS scans from 400 to 1500 m/z were recorded, and the two most intensive peptide ions were subjected to further fragmentation. The MS/MS scans were recorded from 100 to 1500 m/z.

### Protein identification

MS/MS Data was exported using wiff2dta [13], version 1.1.10. Protein identification was performed using Mascot™, Version 2.0 (Matrix Science, London, UK) and the database SwissProt (26-01-2006). Identification data as well as fragment ion spectra were extracted using mres2x [17]. MS/MS peptide identifications were verified using theospec [1] and the visualization tools of resDB [18]. Protein identifications were verified using seqDB [19] as used in former studies [18].

The quantification by ProQuant™ was performed using the Analyst QS™ Software, version 1.1. Proteins were implicitly identified by ProID™ 1.1 using the SwissProt database (26-01-2006). An interrogator database was generated based on the database using the enzyme trypsin and allowing one missed cleavage site. The parameters for ProQuant™ (version 1.1) and Pro Group Report (version 1.0.2) were 1.30 for the protein score threshold, and competitor proteins were shown within a protein score of 2.00. The mass tolerance was set to 0.4 amu for precursor ions and 0.4 amu for fragment ions.

Additionally, Mascot™ 2.2 was used for iTRAQ™ analysis. The protein ratio type was set to median, the normalization method was median ratio, no outlier removal was chosen and the peptide threshold was set to at least homology.

### Error estimation and error propagation

We introduce precise error propagation in quantification software. A common method in error estimation is done by using the mean value *μ*, the standard deviation *σ *and by applying the *kσ*-rule and the Tschebyschew-equation and has been proposed for quantification [12]. But this method implicates the assumption of the independence of the measured values and simultaneously requires their normal distribution (normality). If one of these or both cannot be assured, other means than this statistical approach to error estimation have to be applied. This is the case, if for example each measurement is only made once and uncertainty arises from precision issues of the instruments used. Moreover, the peptide count in quantitative proteomics is not large enough for reliable calculation of a mean and a standard deviation. Then, errors have to be estimated by intervals. The minimum and maximum values are calculated.

Usually in error treatment, observations are denoted with their errors. Let *a *and *b *be two measurements of the true values *a*_0 _and *b*_0 _with the relative errors |*f*_*a*_| and |*f*_*b*_|, respectively. The corresponding absolute errors are denoted as |*e*_*a*_| and |*e*_*b*_|. Then the equations *a *= *a*_0 _(1 ± |*f*_*a*_|) = *a*_0 _± |*e*_*a*_| and *b *= *b*_0 _(1 ± |*f*_*b*_|) = *b*_0 _± |*e*_*b*_| are valid.

Error propagation can be calculated dependant on the mathematical operations as follows. Sum and difference can be estimated as

*a *± *b *∈ {*a*_0 _± *b*_0 _- (|*e*_*a*_| + |*e*_*b*_|), *a*_0 _± *b*_0 _+ (|*e*_*a*_| + |*e*_*b*_|)} and |*e*_*a *± *b*_| = |*e*_*a*_| + |*e*_*b*_|,

and product as well as quotient as

*a*·*b *∈ {*a*_0_·*b*_0_·(1 - (|*f*_*a*_| + |*f*_*b*_|)), *a*_0_·*b*_0_·(1 + (|*f*_*a*_| + |*f*_*b*_|))} and |*f*_*a*·*b*_| = |*f*_*a*_| + |*f*_*b*_|,

ab∈{a0b0(1−(|fa|+|fb|)),a0b0(1+(|fa|+|fb|))}and|fab|=|fa|+|fb|
 MathType@MTEF@5@5@+=feaafiart1ev1aaatCvAUfKttLearuWrP9MDH5MBPbIqV92AaeXatLxBI9gBaebbnrfifHhDYfgasaacH8akY=wiFfYdH8Gipec8Eeeu0xXdbba9frFj0=OqFfea0dXdd9vqai=hGuQ8kuc9pgc9s8qqaq=dirpe0xb9q8qiLsFr0=vr0=vr0dc8meaabaqaciaacaGaaeqabaqabeGadaaakeaafaqabeqadaaabaWaaSqaaSqaaiabdggaHbqaaiabdkgaIbaakiabgIGiopaacmqabaWaaSqaaSqaaiabdggaHnaaBaaameaacqaIWaamaeqaaaWcbaGaemOyai2aaSbaaWqaaiabicdaWaqabaaaaOWaaeWaaeaacqaIXaqmcqGHsisldaqadaqaamaaemaabaGaemOzay2aaSbaaSqaaiabdggaHbqabaaakiaawEa7caGLiWoacqGHRaWkdaabdaqaaiabdAgaMnaaBaaaleaacqWGIbGyaeqaaaGccaGLhWUaayjcSdaacaGLOaGaayzkaaaacaGLOaGaayzkaaGaeiilaWYaaSqaaSqaaiabdggaHnaaBaaameaacqaIWaamaeqaaaWcbaGaemOyai2aaSbaaWqaaiabicdaWaqabaaaaOWaaeWaaeaacqaIXaqmcqGHRaWkdaqadaqaamaaemaabaGaemOzay2aaSbaaSqaaiabdggaHbqabaaakiaawEa7caGLiWoacqGHRaWkdaabdaqaaiabdAgaMnaaBaaaleaacqWGIbGyaeqaaaGccaGLhWUaayjcSdaacaGLOaGaayzkaaaacaGLOaGaayzkaaaacaGL7bGaayzFaaaabaGaeeyyaeMaeeOBa4MaeeizaqgabaWaaqWaaeaacqWGMbGzdaWgaaWcbaWaaSWaaWqaaiabdggaHbqaaiabdkgaIbaaaSqabaaakiaawEa7caGLiWoacqGH9aqpdaabdaqaaiabdAgaMnaaBaaaleaacqWGHbqyaeqaaaGccaGLhWUaayjcSdGaey4kaSYaaqWaaeaacqWGMbGzdaWgaaWcbaGaemOyaigabeaaaOGaay5bSlaawIa7aaaaaaa@7ACC@

This can be applied to the calculation of the determinant of any *m *× *n *matrix M. If any two columns are exchanged, the propagated relative error is not affected. This is especially valid when determinants are calculated by using submatrices.

The absolute error |*e*_*i*_| of the peak intensity *I*_*i *_is 0.5 in case of integer values. In all other cases, this error depends on the precision of the mass spectrometer and must be estimated individually during calibration. An MS/MS spectrum can be defined as a set *M *of 2-tuples *M *= {(*x*_*i*_, *I*_*i*_) | *i *∈ {1,..., *n*}} and the intensities *I*_*i *_can be regarded as error-prone *I*_*i *_= *y*_0 _± |*e*_*i*_| = *y*_0 _(1 ± *f*_*i*_), but derived from the true signal *y*_0_.

### Purity correction of iTRAQ™ labels and error estimation

The iTRAQ™ reagent batches supplied by ABI are provided with sixteen purity values. These indicate the percentages of each reporter ion that have masses differing by -2, -1, +1 and +2 Da from the nominal reporter ion mass due to isotopic variants. Following the method proposed formerly [14], we use this information to correct the values of each reporter ion to account for the losses to and gains from other reporter ions. This results in simultaneous equations that can be framed such that they can be solved by applying Cramer's rule. This is where we extend the published method by means of error propagation. The relative error of the true reporter intensity *W*_*i *_is |fWi|=|fdet⁡(Ci)|=edet⁡(Ci)|det⁡(Ci)|
 MathType@MTEF@5@5@+=feaafiart1ev1aaatCvAUfKttLearuWrP9MDH5MBPbIqV92AaeXatLxBI9gBaebbnrfifHhDYfgasaacH8akY=wiFfYdH8Gipec8Eeeu0xXdbba9frFj0=OqFfea0dXdd9vqai=hGuQ8kuc9pgc9s8qqaq=dirpe0xb9q8qiLsFr0=vr0=vr0dc8meaabaqaciaacaGaaeqabaqabeGadaaakeaadaabdaqaaiabdAgaMnaaBaaaleaacqWGxbWvdaWgaaadbaGaemyAaKgabeaaaSqabaaakiaawEa7caGLiWoacqGH9aqpdaabdaqaaiabdAgaMnaaBaaaleaacyGGKbazcqGGLbqzcqGG0baDcqGGOaakcqWGdbWqdaWgaaadbaGaemyAaKgabeaaliabcMcaPaqabaaakiaawEa7caGLiWoacqGH9aqpdaWcaaqaaiabdwgaLnaaBaaaleaacyGGKbazcqGGLbqzcqGG0baDcqGGOaakcqWGdbWqdaWgaaadbaGaemyAaKgabeaaliabcMcaPaqabaaakeaadaabdaqaaiGbcsgaKjabcwgaLjabcsha0naabmaabaGaem4qam0aaSbaaSqaaiabdMgaPbqabaaakiaawIcacaGLPaaaaiaawEa7caGLiWoaaaaaaa@58A4@, with *i *∈ {114, 115, 116, 117}.

In addition, we introduce an initial experiment error that is taken into consideration during calculation of peptide and especially for protein quantification. In former publications [14], a rough intensity error estimation has been proposed. We improve this by a more reliable estimation. Moreover, our method is not fixed to integer intensity values in the fragment ion spectra.

### Quantification of proteins

**Figure 2:**
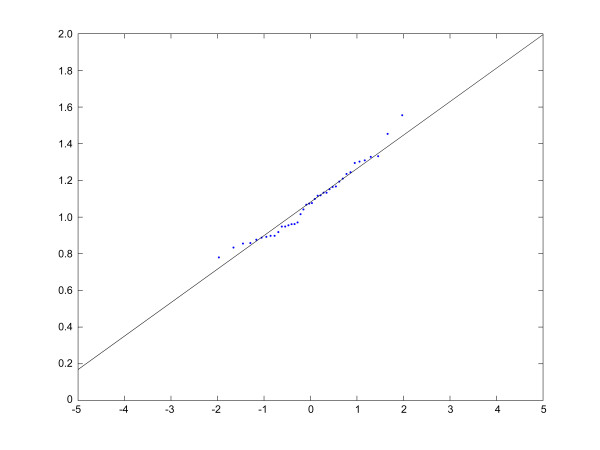
The normal-probability-plot shows that a lognormal distribution fits the peptide ratio data. The transformed experimental data is plotted and lies on a line, so the data is nearly normally distributed. The x-axis denotes the inverse function of the normality and the y-axis represents the sorted log-transformed values.

When performing protein quantification, only unique peptides are taken into consideration, whereas peptides belonging to more than one protein sequence are only used for proving the identification of the corresponding proteins. The ratios of the unique peptides are lognormal distributed if their count *n *is large enough, see figure 2. This has been previously reported for difference gel electrophoresis (DIGE) protein data [20,21]. The Shapiro-Wilk-test, a powerful test of departure from normality, performed with Statistica™ (version 7.1, StatSoft Europe GmbH, Hamburg, Germany) yields W = 0.9629 and a p-value of 0.2095 for the data of the 1:3 mix. Therefore, the null hypothesis that the log-transformed data is normal distributed cannot be rejected due to the high p-value. The median of the ratios is calculated, too. In case of lognormal distribution, this equals the mean value *μ *of the log-transformed and thus normal distributed peptide ratios. However, in case of large *n*, the median should be preferred to the mean value of the non-transformed data, because it represents the medium observation and is thus the more meaningful choice between the both. The median represents the protein ratio. Additionally, the protein ratio *R*_*P *_is calculated using the method of least-squares estimation (LSE) by minimizing the square root ∑i=1n(RP−Ri)2
 MathType@MTEF@5@5@+=feaafiart1ev1aaatCvAUfKttLearuWrP9MDH5MBPbIqV92AaeXatLxBI9gBaebbnrfifHhDYfgasaacH8akY=wiFfYdH8Gipec8Eeeu0xXdbba9frFj0=OqFfea0dXdd9vqai=hGuQ8kuc9pgc9s8qqaq=dirpe0xb9q8qiLsFr0=vr0=vr0dc8meaabaqaciaacaGaaeqabaqabeGadaaakeaadaGcaaqaamaaqahabaWaaeWaaeaacqWGsbGudaWgaaWcbaGaemiuaafabeaakiabgkHiTiabdkfasnaaBaaaleaacqWGPbqAaeqaaaGccaGLOaGaayzkaaWaaWbaaSqabeaacqaIYaGmaaaabaGaemyAaKMaeyypa0JaeGymaedabaGaemOBa4ganiabggHiLdaaleqaaaaa@3C93@. This yields a value with a minimal mean distance from the data points *R*_*i*_. Both, LSE and median represent the protein ration derived from the peptide ratios. The choice of the median as protein ratio bases on the lognormal distribution of the peptide ratios and is a good choice for large enough data sets. The LSE is appropriate for smaller data sets and does not depend on an underlying distribution. This is the average of the points Ri, as can be shown. Both values should be nearly equal and their difference can be regarded as an additional quality measure. Moreover, if the peptide ratio count is large enough, the mean value *μ *and standard deviation *σ *of the log-transformed peptide ratios can be used as quality indicators, too.

### Implementation

The implementation was done on MATLAB^® ^(The Mathworks, Ismaning, Germany), version 6.1. The program files are contained in additional file 1, the detailed documentation in additional file 2. We provide example data in additional file 3.

The quantification values are calculated by the script startquantitraq. It executes quantitraq that performs the iTRAQ™ quantification. The integration is done by calling sumquantitraq (sum of intensities) or flquantitraq (area calculation by trapezoids), depending on the user's choice. The function pcquantitraq implements the purity correction and is called by quantitraq. The peptide ratios are calculated by raquantitraq. The list of files being processed in batch is provided in the file names01.txt. These files contain the uncentroided MS/MS spectra in DTA format. We recommend not using centroided MS/MS spectra. Mascot™ results could be exported by using mres2x [17], for instance. The script startexperror performs the calculation of the experiment error by execution of the functions experror that calls logtrans, qplot and killzero. By running startplotitraq, the errors are plotted and the boxplots are created by iteratively calling plotitraq. The result files listed in the file names02.txt are processed.

## Results

### Peptide quantification based on fragment ion spectra

In contrast to other quantification software such as i-Tracker [14] or RelEx [22], Quant is able to cope with just one signal per iTRAQ™ reporter ion. We allow the choice between two methods of integration: trapezoid integration as implemented in existing software tools and the sum of intensities (see below). We introduce a constant minimal peak width *b *that is applied if only one peak is found in order to allow calculation of a peak area *A *when trapezoid integration has been chosen. The error estimation in the former case is as follows: |*f*_*A*_| = |*f*_*i*_| ⇒ *e*_*A *_= |*e*|·*b*. In the latter case, the absolute error of trapezoid integration of peaks {(*x*_*i*_, *y*_*i*_)} belonging to the mass spectrum *S *= {(*x*_1_, *y*_1_),...,(*x*_*n*_, *y*_*n*_)} is |*e*_*A*_| = |*e*|·(*x*_*n *_- *x*_1_). The absolute error when summing up the intensities is |*e*_*S*_| = *n*·|*e*|.

Relative quantification is performed by calculation of peptide ratios. Each pair of ratios is calculated by building quotients *R*_*i*, *j *_of the true reporter intensities *W*_*i *_and *W*_*j*_, based on area or sum, for example R114,115=W114W115
 MathType@MTEF@5@5@+=feaafiart1ev1aaatCvAUfKttLearuWrP9MDH5MBPbIqV92AaeXatLxBI9gBaebbnrfifHhDYfgasaacH8akY=wiFfYdH8Gipec8Eeeu0xXdbba9frFj0=OqFfea0dXdd9vqai=hGuQ8kuc9pgc9s8qqaq=dirpe0xb9q8qiLsFr0=vr0=vr0dc8meaabaqaciaacaGaaeqabaqabeGadaaakeaacqWGsbGudaWgaaWcbaGaeGymaeJaeGymaeJaeGinaqJaeiilaWIaeGymaeJaeGymaeJaeGynaudabeaakiabg2da9maalaaabaGaem4vaC1aaSbaaSqaaiabigdaXiabigdaXiabisda0aqabaaakeaacqWGxbWvdaWgaaWcbaGaeGymaeJaeGymaeJaeGynaudabeaaaaaaaa@3E31@. Consequently, the implicated relative error of the quotient is |fRi,j|=|fWi|+|fWj|
 MathType@MTEF@5@5@+=feaafiart1ev1aaatCvAUfKttLearuWrP9MDH5MBPbIqV92AaeXatLxBI9gBaebbnrfifHhDYfgasaacH8akY=wiFfYdH8Gipec8Eeeu0xXdbba9frFj0=OqFfea0dXdd9vqai=hGuQ8kuc9pgc9s8qqaq=dirpe0xb9q8qiLsFr0=vr0=vr0dc8meaabaqaciaacaGaaeqabaqabeGadaaakeaadaabdaqaaiabdAgaMnaaBaaaleaacqWGsbGudaWgaaadbaGaemyAaKMaeiilaWIaemOAaOgabeaaaSqabaaakiaawEa7caGLiWoacqGH9aqpdaabdaqaaiabdAgaMnaaBaaaleaacqWGxbWvdaWgaaadbaGaemyAaKgabeaaaSqabaaakiaawEa7caGLiWoacqGHRaWkdaabdaqaaiabdAgaMnaaBaaaleaacqWGxbWvdaWgaaadbaGaemOAaOgabeaaaSqabaaakiaawEa7caGLiWoaaaa@472E@, the absolute error |eRi,j|=|fRi,j|Ri,j
 MathType@MTEF@5@5@+=feaafiart1ev1aaatCvAUfKttLearuWrP9MDH5MBPbIqV92AaeXatLxBI9gBaebbnrfifHhDYfgasaacH8akY=wiFfYdH8Gipec8Eeeu0xXdbba9frFj0=OqFfea0dXdd9vqai=hGuQ8kuc9pgc9s8qqaq=dirpe0xb9q8qiLsFr0=vr0=vr0dc8meaabaqaciaacaGaaeqabaqabeGadaaakeaadaabdaqaaiabdwgaLnaaBaaaleaacqWGsbGudaWgaaadbaGaemyAaKMaeiilaWIaemOAaOgabeaaaSqabaaakiaawEa7caGLiWoacqGH9aqpdaabdaqaaiabdAgaMnaaBaaaleaacqWGsbGudaWgaaadbaGaemyAaKMaeiilaWIaemOAaOgabeaaaSqabaaakiaawEa7caGLiWoacqWGsbGudaWgaaWcbaGaemyAaKMaeiilaWIaemOAaOgabeaaaaa@45F5@.

**Figure 3:**
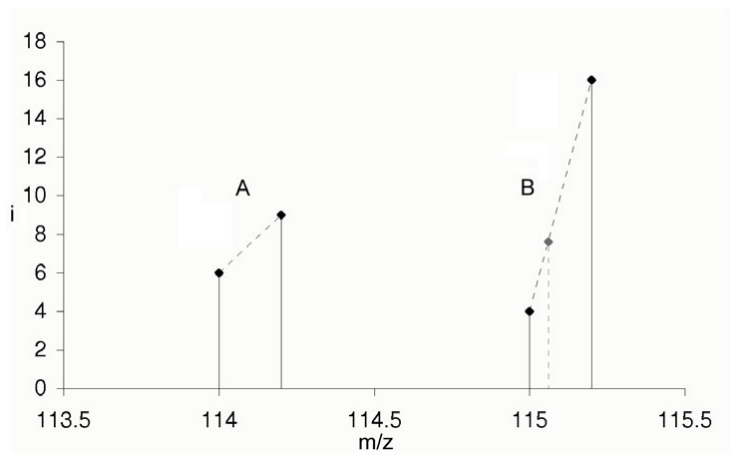
The example peaks of two labels A and B are depicted. The area of the peaks is not proportional to the sum of intensities if peak distances and peak count are not equal. This has effects on the quantification results yielding notable differences. The summed intensities of the example above are 15.0000 and 20.000, respectively. The trapezoid integrals amount to 1.500 (A) and 2.000 (B). The corresponding ratios are 1.3333 (summed) and 1.3333 (area). Suppose an additional peak at 115.0600 m/z with an intensity of 7.6000. Then, the area of B would be the identical, whereas the summed intensities will change to 27.6000, yielding a ratio of 1.8400. This yields a difference in relative quantification of 38%. In the former case, the ratio would not reflect the ion count of the three peaks detected by the mass spectrometer, but the latter does as the intensity of each signal represents the amount of ions detected and counted by the mass spectrometer.

The effects of the chosen integration method are as follows. The quadratic effect of the integration process that comes from the area calculation does not disappear by applying quotients when ratios are calculated. Consider the example of two labels with two peaks each: *P*_*A *_= {(114.0000, 6.0000), (114.2000, 9.0000)} (label A) and *P*_*B *_= {(115.0000, 4.0000), (115.2000, 16.0000)} (label B), see figure 3. The summed intensities are 15.0000 and 20.0000, respectively. The trapezoid integrals amount to 1.5000 (A) and 2.0000 (B). The corresponding ratios are 1.3333 (summed) and 1.3333 (area). If an additional peak would have been acquired at for example 115.0600 m/z with an intensity of 7.6000, the area of B will not change, but the summed intensity will change to 27.6000, yielding a ratio of 1.8400. This yields a difference in relative quantification of about 38%. Therefore, we recommend using the sum of intensities instead of calculating an underlying area.

These distorting effects of the integration method are independent of the peak-picking method (centroid, gaussian peak detection etc.) that is applied by the data extraction software processing the raw data of the mass spectrometer. Quant itself uses MS/MS data extracted by other means and therefore is independent of any peak-picking method. Moreover it is independent of the mass spectrometer manufacturer and of the controlling software.

Quant integrates an "experiment error" for protein quantification, i.e. a shift of peptide ratios that indicates the overall protein quantification. Previous studies have shown by plotting the ratio distribution of the proteins that most proteins of a sample are not regulated [23,24]. Therefore, the distribution of peptide ratios obtained by a quantification experiment should scatter around a value of one. If this is not the case, this shift indicates an error that happened during the sample preparation in the laboratory. Consider the example of mixing two samples 1:1. The protein concentration has to be known. This can be determined by a BCA [25,26] or Bradford assay [27], but both are not precise as other colorimetric protein assays, too [28]. Thus no exact 1:1 mix can be guaranteed during sample preparation.

Moreover, errors could occur during pipetting, particularly when handling small amounts of protein sample. In order to quantify this shift, the distribution of the peptide ratios must be analyzed in detail.

Firstly, the type of distribution must be determined. We found all peptide ratios lognormal distributed as reported previously for DIGE protein data [20,21]. The median was chosen as parameter, because the log-transformed median equals the mean of the log-transformed normal distributed data. Besides the observation, that biological data mostly are lognormal distributed, in the case of peptide quantification a left-steeply, right skewed distribution is observed. This can be explained by the fact that in peptide quantification, the ratios have values greater than zero, but very seldom large values. Usually, they vary around 1. The lognormal distribution can be proved by a normal-probability-plot as shown in figure 2.

The definition of the median in conjunction with the multiplicative characteristic of the lognormal distribution implies that the shift in question is multiplicative, too. This factor is the reciprocal of the median. All peptide ratios are multiplied with this value. Consequently, the median of the shifted peptide ratios is then near one.

The multiplication of the ratios with the median *m *effects the error estimation. The absolute error changes from *e*_*R *_to eR′=1meR
 MathType@MTEF@5@5@+=feaafiart1ev1aaatCvAUfKttLearuWrP9MDH5MBPbIqV92AaeXatLxBI9gBaebbnrfifHhDYfgasaacH8akY=wiFfYdH8Gipec8Eeeu0xXdbba9frFj0=OqFfea0dXdd9vqai=hGuQ8kuc9pgc9s8qqaq=dirpe0xb9q8qiLsFr0=vr0=vr0dc8meaabaqaciaacaGaaeqabaqabeGadaaakeaacqWGLbqzdaWgaaWcbaGafmOuaiLbauaaaeqaaOGaeyypa0ZaaSaaaeaacqaIXaqmaeaacqWGTbqBaaGaemyzau2aaSbaaSqaaiabdkfasbqabaaaaa@3583@. The relative error *f*_*m *_of *m *implies a relative error of *f *= *f*_*R *_+ *f*_*m *_when calculating the quotients.

Multiple labelling of peptides has no effects on the quantification results, because the peptides being compared have identical sequences, and thus are equally labelled.

### Protein quantification and visualization

The in-house implementation of a pipeline that integrates Quant accepts peptide identifications from either Mascot™ [29] or Sequest™ [30] and integrates the tool mres2x [17] in order to preserve the linkage between the peptide identification and the corresponding MS/MS spectra.

**Figure 4:**
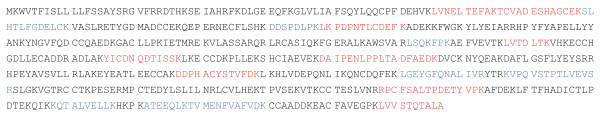
The amino acid sequence of the protein bovine serum albumin (P02769) is depicted as an example for sequence coverage. The uniquely identified peptide sequences are marked in red, whereas the blue marked regions are confirmed by non-unique peptides. The sequence coverage of the example shown above is 33.61%, the covered mass is 33.27%.

Usually, only unique peptides are taken into consideration, whereas peptides pointing to more than one protein sequence are only used for improving protein identification as well as for verification and confirmation of identifications (see figure 4).

**Figure 5:**
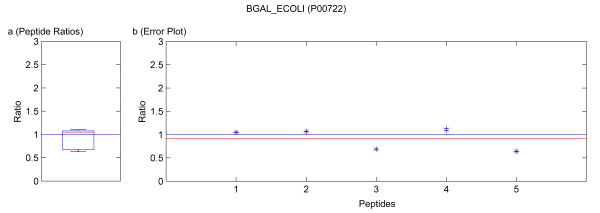
Quantification results of the protein BGAL_ECOLI (P00722). Samples were mixed in a ratio of 1:1. Figure **a) **shows the standard boxplot of the peptide ratios. The median is 1.0508. Figure **b) **depicts the protein ratio calculated by the LSE value of the single peptide ratios, 0.9106. The red line indicates the LSE value, i.e. the protein ratio calculated from the relative peptide abundances. The blue crosses mark the corresponding errors of each peptide ratio, the red ones the peptide ratios.

**Figure 6:**
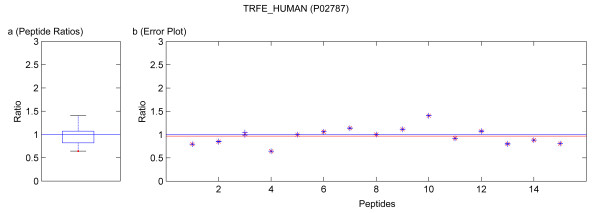
Quantification results of the protein TRFE_HUMAN (P02787). Samples were mixed in a ratio of 1:1. Figure **a) **shows the standard boxplot of the peptide ratios. The median is 0.9984. Outliers are marked in red. Figure **b) **depicts the protein ratio calculated by the LSE value of the single peptide ratios, 0.9673. The red line indicates the LSE value, i.e. the protein ratio calculated from the relative peptide abundances. The blue crosses mark the corresponding errors of each peptide ratio, the red ones the peptide ratios.

**Figure 7:**
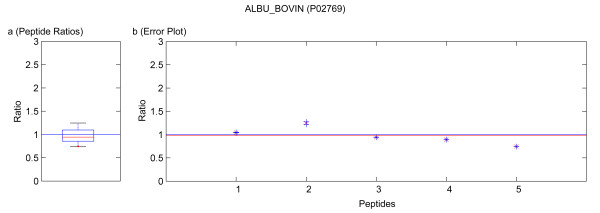
Quantification results of the protein ALBU_BOVIN (P02769). Samples were mixed in a ratio of 1:3. Figure **a) **shows the standard boxplot of the peptide ratios. The median is 0.9424. Figure **b) **depicts the protein ratio calculated by the LSE value of the single peptide ratios, 0.9742. The red line indicates the LSE value, i.e. the protein ratio calculated from the relative peptide abundances. The blue crosses mark the corresponding errors of each peptide ratio, the red ones the peptide ratios.

**Figure 8:**
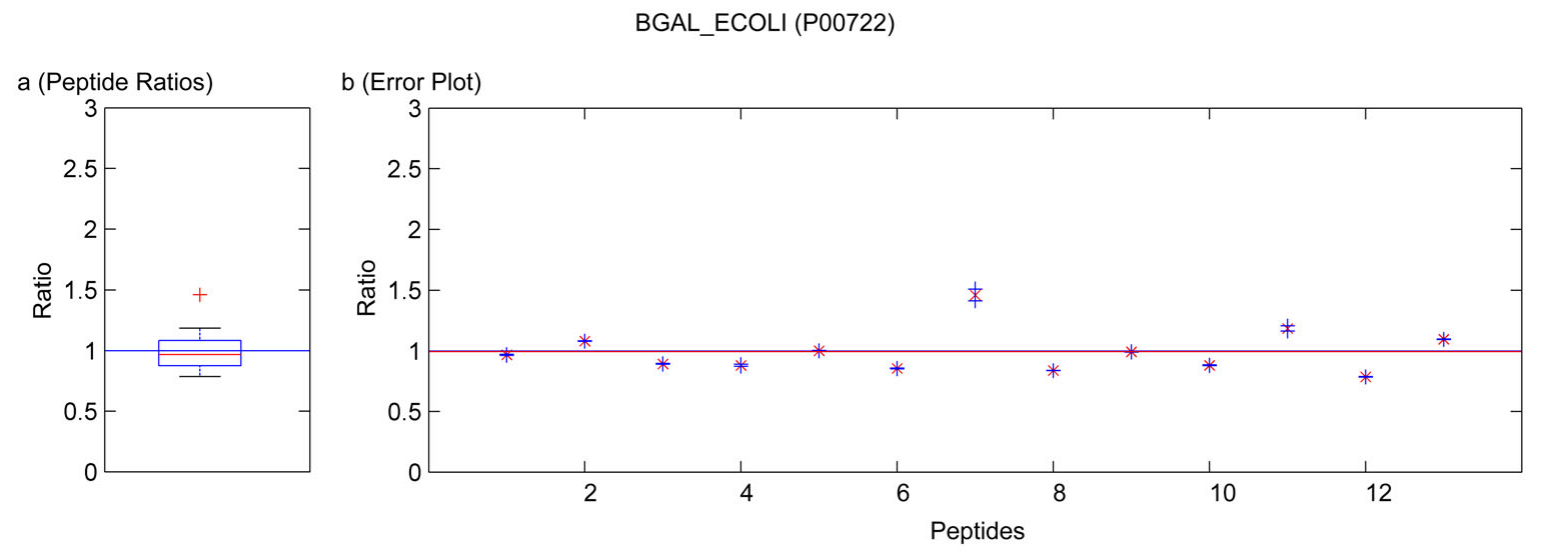
Quantification results of the protein BGAL_ECOLI (P00722). Samples were mixed in a ratio of 1:3. The sequence of the outlying peptide 7 is APLDNDIGVSEATR with a ratio of 1.5171 ± 0.0121. Figure **a) **shows the standard boxplot of the peptide ratios. The median is 0.9674. Outliers are marked in red. They not distort the calculation of the protein ratio. Figure **b) **depicts the protein ratio calculated by the LSE value of the single peptide ratios, 0.9936. The red line indicates the LSE value, i.e. the protein ratio calculated from the relative peptide abundances. The blue crosses mark the corresponding errors of each peptide ratio, the red ones the peptide ratios.

**Figure 9:**
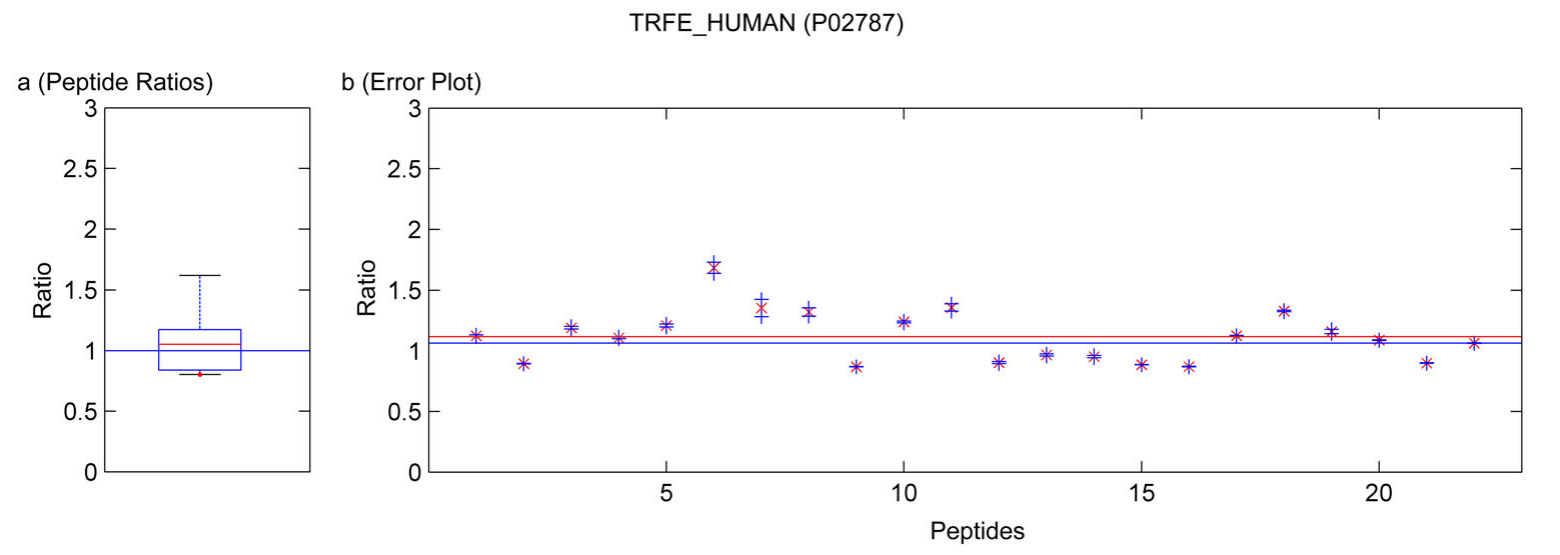
Quantification results of the protein TRFE_HUMAN (P02787). Samples were mixed in a ratio of 1:3. Figure **a) **shows the standard boxplot of the peptide ratios. The median is 1.0511. Figure **b) **depicts the protein ratio calculated by the LSE value of the single peptide ratios, 1.0526. The red line indicates the LSE value, i.e. the protein ratio calculated from the relative peptide abundances. The blue crosses mark the corresponding errors of each peptide ratio, the red ones the peptide ratios.

Visualization of protein quantification is done by providing a boxplot of the peptide ratios, as depicted in figures 5, 6, 7, 8, 9. This includes the first and third quartile of the data, i.e. the 25% and 75% quantile. The median is depicted by a horizontal red line. The whiskers mark the data range and are limited to 150% of the inter-quartile-range (IQR). Outliers are marked in red. The IQR represents a quality measure as it quantifies the scatter of the data independent of the underlying distribution.

As a measure of quality, the confidence interval [*μ*-*k*·*σ*, *μ *+ *k*·*σ*] can be used for the log-transformed data. Additionally, the standard deviation *σ *of this data can be used as an indicator of the quantification quality in case of a large peptide count per protein. As a numeric tool for measuring the overall quality of the data used, the root-mean-square value (RMS) can be applied to the relative errors of peptide quantification: RMS(f→)=1n∑i=1nfi2=|f→|n
 MathType@MTEF@5@5@+=feaafiart1ev1aaatCvAUfKttLearuWrP9MDH5MBPbIqV92AaeXatLxBI9gBaebbnrfifHhDYfgasaacH8akY=wiFfYdH8Gipec8Eeeu0xXdbba9frFj0=OqFfea0dXdd9vqai=hGuQ8kuc9pgc9s8qqaq=dirpe0xb9q8qiLsFr0=vr0=vr0dc8meaabaqaciaacaGaaeqabaqabeGadaaakeaacqWGsbGucqWGnbqtcqWGtbWudaqadaqaaiqbdAgaMzaalaaacaGLOaGaayzkaaGaeyypa0ZaaOaaaeaadaWcbaWcbaGaeGymaedabaGaemOBa4gaaOWaaabCaeaacqWGMbGzdaqhaaWcbaGaemyAaKgabaGaeGOmaidaaaqaaiabdMgaPjabg2da9iabigdaXaqaaiabd6gaUbqdcqGHris5aaWcbeaakiabg2da9maaleaaleaadaabdaqaaiqbdAgaMzaalaaacaGLhWUaayjcSdaabaWaaOaaaeaacqWGUbGBaWqabaaaaaaa@48A9@.

The smaller the RMS-value, the better the level of uncertainty is. This method must be preferred to the norm of an error vector, because the dimensions of error vectors are not identical. Moreover, the RMS is appropriate for small data sets.

### Experimental results

The standard protein mix supplied with the iTRAQ™ kit was used for testing our software tool Quant. The contents and amount of proteins are known and this protein mix is generally used to establish the iTRAQ™ workflow in laboratories.

Furthermore, we always test new software with a generalized and known sample. By doing this, the functionality and applicability can be easily shown.

The standard protein mix of iTRAQ™ consists of bovine serum albumin (*Bos taurus*), β-galactosidase (*E. coli*), α-lactalbumin (*Bos taurus*), β-lactoglobulin (*Bos taurus*), lysozyme (*Gallus gallus*), apo-transferrin (*Homo sapiens*). The results acquired by following our standardised protein identification procedure that comprises LC-MS/MS and the database search algorithm Mascot™ 2.0 are shown in tables 1 and 2. These data prove that all expected proteins have been identified. However, several homologous proteins are detected, because the complete database SwissProt was used for identification. In the tables 1 and 2, all peptides belonging to more than one protein are marked in red. To visualize the unique and non-unique peptides of a protein, an example is shown in figure 4.

**Table 1:** Identified proteins of the 1:1 sample mix

**Protein Id**	**Protein name**	**Mascot™ score (#_i_, #_q_)**	**Peptide sequences**	**Identical set of peptides**
P49663	Lysozyme C LYSC_PHAVE	797 (4, 1)	CELAAAMK (*) IVSDGDGMNAWVAWR (*) KIVSDGDGMNAWVAWR NTDGSTDYGILQINSR (*)	

P00698	Lysozyme C precursor LYSC_CHICK	655 (5, 3)	CELAAAMK (*) FESNFNTQATNR GTDVQAWIR KIVSDGNGMNAWVAWR NTDGSTDYGILQINSR (*)	

P00722	Beta-galactosidase BGAL_ECOLI	556 (17, 17)	APLDNDIGVSEATR DWENPGVTQLNR GDFQFNISR IDPNAWVER IGLNCQLAQVAER LNVENPK LSGQTIEVTSEYLFR QSGFLSQMWIGDKK RDWENPGVTQLNR VDEDQPFPAVPK VNWLGLGPQENYPDR VVQPNATAWSEAGHISAWQQWR WSDGSYLEDQDMWR WVGYGQDSR YDENGNPWSAYGGDFGDTPNDR YGLYVVDEANIETHGMVPMNR YSQQQLMETSHR	

P02787	Serotransferrin precursor TRFE_HUMAN	481 (19, 19)	ADRDQYELLCLDNTR DCHLAQVPSHTVVAR DDTVCLAK DGAGDVAFVK DSAHGFLK EDPQTFYYAVAVVK EGYYGYTGAFR FDEFFSEGCAPGSK HQTVPQNTGGK KPLEK KPVEEYANCHLAR KSASDLTWDNLK MYLGYEYVTAIR NLNEKDYELLCLDGTR NPDPWAK SASDLTWDNLK SDNCEDTPEAGYFAVAVVK SVIPSDGPSVACVK TAGWNIPMGLLYNK	

P02769	Serum albumin precursor ALBU_BOVIN	391 (19, 10)	ATEEQLK (*) DAIPENLPPLTADFAEDK DDPHACYSTVFDK DDSPDLPK (*) DLGEEHFK KQTALVELLK (*) KVPQVSTPTLVEVSR (*) LGEYGFQNALIVR (*) LKPDPNTLCDEFK LSQKFPK (*) LVNELTEFAK LVTDLTK LVVSTQTALA RPCFSALTPDETYVPK SLHTLFGDELCK (*) TCVADESHAGCEK TVMENFVAFVDK (*) VPQVSTPTLVEVSR (*) YICDNQDTISSK	

P00706	Lysozyme C-3 LYSC3_ANAPL	333 (3, 1)	CELAAAMK (*) IVSDGDGMNAWVAWR (*) NTDGSTDYGILEINSR	

P49064	Serum albumin precursor ALBU_FELCA	169 (5, 0)	ATEEQLK (*) KVPQVSTPTLVEVSR (*) LGEYGFQNALLVR (*) LSQKFPK (*) VPQVSTPTLVEVSR (*)	

P02754	Beta-lactoglobulin precursor LACB_BOVIN	169 (6, 0)	IPAVFK (*) TKIPAVFK (*) TPEVDDEALEK (*) TPEVDDEALEKFDK (*) VLVLDTDYK (*) VLVLDTDYKK (*)	LACB_BUBBU

P49822	Serum albumin precursor ALBU_CANFA	115 (2, 0)	KQTALVELLK (*) LGEYGFQNALLVR (*)	

P07724	Serum albumin precursor ALBU_MOUSE	104 (1, 0)	LGEYGFQNAILVR (*)	

P14639	Serum albumin precursor ALBU_SHEEP	96 (6, 1)	DDSPDLPK (*) IVTDLTK KQTALVELLK (*) LSQKFPK (*) SLHTLFGDELCK (*) TVMENFVAFVDK (*)	

P00711	Alpha-lactalbumin precursor LALBA_BOVIN	88 (1, 0)	VGINYWLAHK (*)	LALBA_BOSMU LALBA_BUBBU LALBA_CAPHI LALBA_SHEEP

Peptides belonging to more than one protein are marked with an asterisk (*). Proteins mentioned in the right column are confirmed by an identical set of peptides. Reliable quantification can only be performed on unique peptides whose spectra contain reporter signals. (#_i_: number of identified peptides, #_q_: number of peptides for quantification)

**Table 2:** Identified proteins of the 1:3 sample mix.

**Protein Id**	**Protein name**	**Mascot™ score (#_i_, #_q_)**	**Peptide sequences**	**Identical identification**
P00722	Beta-galactosidase BGAL_ECOLI	746 (15, 15)	APLDNDIGVSEATR DWENPGVTQLNR GDFQFNISR IDPNAWVER LSGQTIEVTSEYLFR QNNFNAVR QSGFLSQMWIGDK QSGFLSQMWIGDKK RDWENPGVTQLNR VNWLGLGPQENYPDR VVQPNATAWSEAGHISAWQQWR WSDGSYLEDQDMWR WVGYGQDSR YGLYVVDEANIETHGMVPMNR YSQQQLMETSHR	

P49663	Lysozyme C (LYSC_PHAVE	440 (5, 1)	CELAAAMK (*) GYSLGNWVCAAK (*) IVSDGDGMNAWVAWR (*) KIVSDGDGMNAWVAWR NTDGSTDYGILQINSR (*)	

P00698	Lysozyme C precursor LYSC_CHICK	368 (7, 3)	CELAAAMK (*) FESNFNTQATNR GTDVQAWIR GYSLGNWVCAAK (*) IVSDGNGMNAWVAWR (*) KIVSDGNGMNAWVAWR NTDGSTDYGILQINSR (*)	

P02787	Serotransferrin precursor TRFE_HUMAN	343 (16, 16)	ADRDQYELLCLDNTR DCHLAQVPSHTVVAR DDTVCLAK DSAHGFLK EDPQTFYYAVAVVK EFQLFSSPHGK EGYYGYTGAFR FDEFFSEGCAPGSK HQTVPQNTGGK KPVEEYANCHLAR LCMGSGLNLCEPNNK MYLGYEYVTAIR NPDPWAK SVIPSDGPSVACVK TAGWNIPMGLLYNK WCAVSEHEATK	

Q7LZQ2	Lysozyme C LYSC_AIXSP	248 (4, 0)	CELAAAMK (*) GYSLGNWVCAAK (*) IVSDGNGMNAWVAWR (*) NTDGSTDYGILEINSR (*)	

P02769	Serum albumin precursor ALBU_BOVIN	206 (16, 10)	ATEEQLK (*) CCTESLVNR (*) DAFLGSFLYEYSR DDPHACYSTVFDK DDSPDLPK ECCHGDLLECADDR (*) ETYGDMADCCEK HLVDEPQNLIK KQTALVELLK (*) LCVLHEK (*) LGEYGFQNALIVR (*) LKPDPNTLCDEFK LVVSTQTALA TCVADESHAGCEK TVMENFVAFVDK YICDNQDTISSK	

P00706	Lysozyme C-3 LYSC3_ANAPL	174 (3, 0)	CELAAAMK (*) IVSDGDGMNAWVAWR (*) NTDGSTDYGILEINSR (*)	

P49064	Serum albumin precursor ALBU_FELCA	69 (5, 0)	ATEEQLK (*) CCTESLVNR (*) ECCHGDLLECADDR (*) LCVLHEK (*) LGEYGFQNALLVR (*)	

P49822	Serum albumin precursor ALBU_CANFA	62 (4, 0)	ECCHGDLLECADDR (*) KQTALVELLK (*) LCVLHEK (*) LGEYGFQNALLVR (*)	

P07724	Serum albumin precursor ALBU_MOUSE	62 (3, 1)	ECCHGDLLECADDR (*) LGEYGFQNAILVR (*) VCLLHEK	

P00711	Alpha-lactalbumin precursor LALBA_BOVIN	49 (1, 0)	VGINYWLAHK (*)	LALBA_BOSMU LALBA_BUBBU LALBA_CAPHI LALBA_SHEEP

P02754	Beta-lactoglobulin precursor LACB_BOVIN	49 (3, 0)	IPAVFK (*) TKIPAVFK (*) TPEVDDEALEK (*)	LACB_BUBBU

Peptides belonging to more than one protein are marked with an asterisk (*). Proteins mentioned in the right column are confirmed by an identical set of peptides. Reliable quantification is only possible by using unique peptides for analysis whose spectra contain reporter signals. (#_i_: number of identified peptides, #_q_: number of peptides for quantification)

The list of identification was then submitted to quantification by Quant. As only unique peptides can be used for reliable quantification, the software Quant implements a filter that removes all non-unique peptides. In a real non-standard sample this is necessary as otherwise protein isoforms neither can be distinguished correctly nor quantified in a reliable manner (see tables 3 and 4 as well as tables 5 and 6, respectively).

**Table 3:** Quantification results of the sample 1:1 mix.

**Protein Id**	**Protein name**	**Quant**						**EE**	**Pro Quant™**			**Bias**
												
								**1.3451**				**1.1439**
		
		**Ratio**	**n**	**IQR**	**RMS**	**LSE**	**μ**	**σ**	**Ratio**	**n**	**pVal**	**EF**
P00698	Lysozyme C precursor LYSC_CHICK	0.7028	23	0.1581	0.0599	0.7973	-0.3169	0.4005	0.9244	23	0.3050	1.1668
P00722	Beta-galactosidase BGAL_ECOLI	0.8128	47	0.1902	0.0227	0.8268	-0.2365	0.3160	0.9256	47	0.2604	1.1455
P02787	Serotransferrin precursor TRFE_HUMAN	1.0053	118	0.6406	0.0453	1.2809	0.1111	0.4776	1.2401	118	0.0007	1.1291
P02769	Serum albumin precursor ALBU_BOVIN	0.8441	93	0.3619	0.0590	1.0420	-0.1225	0.5938	1.1436	93	0.0247	1.1224
P02754	Beta-lactoglobulin precursor LACB_BOVIN	1.3504	57	1.3201	0.0114	1.3495	0.0851	0.7473	1.1864	57	0.3423	1.4382
P00711	Alpha-lactalbumin precursor LALBA_BOVIN	0.7280	13	0.2355	0.0049	0.9227	-0.1637	0.3698	1.0557	13	0.7563	1.4874

The quantification results of the sample 1:1 mix include all peptides of each identified protein. Listed are the Protein Id, the protein name, the protein ratio (ratio) with the experiment error (EE) and the bias estimate of Quant and ProQuant™, respectively. The number of MS/MS spectra is listed in column n. Furthermore, several quality factors calculated by Quant are shown: the least squares estimator (LSE), interquartile range (IQR), root-mean-square value (RMS), mean value (μ) and standard deviation (σ). μ and σ were calculated from the log-transformed data. The results of ProQuant™ are taken from the ProGroup™ output that yields a p-value (pVal) and an error factor (EF) as described previously [35]. The experiment error of Quant (bias of ProQuant™) indicates the overall protein mixing ratio. The protein ratio results are normalized by this factor.

**Table 4:** Quantification results of the sample 1:1 mix.

**Protein Id**	**Protein name**	**Quant**						**EE**	**Mascot™ 2.2**	
										
								**1.4126**		
		
		**Ratio**	**n**	**IQR**	**RMS**	**LSE**	**μ**	**σ**	**Ratio**	**σ**
P00722	Beta-galactosidase BGAL_ECOLI	1.0508	5	0.4003	0.0104	0.9106	-0.1207	0.2656	0.9600	NN
P02787	Serotransferrin precursor TRFE_HUMAN	0.9984	15	0.2522	0.0076	0.9673	-0.0500	0.1902	1.1660	NN

The quantification results of the sample 1:1 mix are shown that include all unique peptides of each identified protein. Listed are the Protein Id, the protein name, and the protein ratio (ratio) with the experiment error (EE) estimate of Quant. The number of MS/MS spectra is listed in column n. Several quality factors calculated by Quant are shown: the least squares estimator (LSE), interquartile range (IQR), root-mean-square value (RMS), mean value (μ) and standard deviation (σ). μ and σ of Quant results were calculated from the log-transformed data. For comparison, results of Mascot™ 2.2 are listed. The protein ratios of the results are normalized.

**Table 5:** Quantification results of the sample 1:3 mix.

**Protein Id**	**Protein name**	**Quant**						**EE**	**Pro Quant™**			**Bias**
												
								**2,9929**				**2,8491**
		
		**Ratio**	**n**	**IQR**	**RMS**	**LSE**	**μ**	**σ**	**Ratio**	**n**	**pVal**	**EF**
P00722	Beta-galactosidase BGAL_ECOLI	1.0072	100	0.1606	0.0287	1.0550	0.0323	0.1952	0.9862	100	0.3045	1.0268
P00698	Lysozyme C precursor LYSC_CHICK	1.0545	47	0.1971	0.0202	1.0891	0.0757	0.1392	1.0088	47	0.6644	1.0410
P02787	Serotransferrin precursor TRFE_HUMAN	1.0005	124	0.1832	0.0213	1.0363	0.0144	0.1973	1.0261	124	0.0536	1.0264
P02769	Serum albumin precursor ALBU_BOVIN	1.0305	64	0.1975	0.0331	1.0929	0.0624	0.2183	1.0329	64	0.0099	1.0245
P00711	Alpha-lactalbumin precursor LALBA_BOVIN	0.9618	8	0.1667	0.0083	0.9546	-0.0511	0.1022	1.0194	8	0.5019	1.0601
P02754	Beta-lactoglobulin precursor (LACB_BOVIN)	1.0366	54	0.1569	0.0099	1.0436	0.0331	0.1388	0.9839	54	0.4429	1.0434

The quantification results of the sample 1:3 mix are shown that include all peptides of each identified protein. Listed are the Protein Id, the protein name, the protein ratio (ratio) with the experiment error (EE) and the bias estimate of Quant and ProQuant™, respectively. The number of MS/MS spectra is listed in column n. Furthermore, several quality factors calculated by Quant are shown: the least squares estimator (LSE), interquartile range (IQR), root-mean-square value (RMS), mean value (μ) and standard deviation (σ). μ and σ were calculated from the log-transformed data. The results of ProQuant™ are taken from the ProGroup™ output that yields a p-value (pVal) and an error factor (EF) as described previously [35]. The experiment error of Quant (bias of ProQuant™) indicates the overall protein mixing ratio. The protein ratio results are normalized by this factor.

**Table 6:** Quantification results of the sample 1:3 mix.

**Protein Id**	**Protein name**	**Quant**						**EE**	**Mascot™ 2.2**	
										
								**2.9306**		
		
		**Ratio**	**n**	**IQR**	**RMS**	**LSE**	**μ**	**σ**	**Ratio**	**σ**
P02769	Serum albumin precursor ALBU_BOVIN	0.9424	5	0.2392	0.0100	0.9742	-0.0407	0.1906	0.9810	1.1000
P00722	Beta-galactosidase BGAL_ECOLI	0.9674	13	0.2101	0.0113	0.9936	-0.0203	0.1687	0.9910	NN
P02787	Serotransferrin precursor TRFE_HUMAN	1.0511	22	0.3356	0.0168	1.0526	0.0332	0.1924	0.9810	NN

The quantification results of the sample 1:3 mix are shown that include all unique peptides of each identified protein. Listed are the Protein Id, the protein name, and the protein ratio (ratio) with the experiment error (EE) estimate of Quant. The number of MS/MS spectra is listed in column n. Several quality factors calculated by Quant are shown: the least squares estimator (LSE), interquartile range (IQR), root-mean-square value (RMS), mean value (μ) and standard deviation (σ). μ and σ of Quant results were calculated from the log-transformed data. For comparison, results of Mascot™ 2.2 are listed. The protein ratios of the results are normalized.

Running the software Quant with this filter, only quantification results for the proteins BGAL_ECOLI, TRFE_HUMAN, ALBU_BOVIN were calculated as for the other proteins only non-unique peptides were detected. These quantification results are presented in tables 4 and 6. In the case of using a known standard protein mix with proteins from different organisms, the non-unique peptides are accessible by deactivation of that filter. This can be avoided in a real sample because the organism is usually known and the database search can be accomplished with a database only containing the proteins of this organism or by using a taxonomy filter as supported by Mascot™. The quantification results obtained by not applying the filter for unique and non-unique peptides are summarized in tables 4, 6 and 3, 5, respectively. In these tables not only the results from our software Quant are listed, but additionally the output of the software ProQuant™ that implements no restriction to only unique peptides. Data obtained from Mascot™ 2.2 are presented, too. The absolute protein quantification ratios yielded by Quant, Mascot™ 2.2, and ProQuant™ are comparable. As shown in tables 3 and 5, including non-unique peptides distorts the quantification results. The experiment error of Quant (bias of ProQuant™) indicates the overall protein mixing ratio. The protein ratio results are normalized by this factor. The visualization of the protein results for BGAL_ECOLI, TRFE_HUMAN and ALBU_BOVIN is shown in figures 5, 6, 7, 8, 9. No peptides were detected that underwent N-terminal cyclation.

## Discussion

### Comparison with other software tools

In contrast to other software used for peptide quantification that applies trapezoid or other methods of integration for area calculation, we decided to introduce the sum of intensities in MS-based quantification. We have shown that integration implies changes in relative quantification of peptides and proteins, see figure 3. This yields similar changes when absolute quantification is performed. The effect depends on the precision, resolution and calibration of the mass spectrometer, but is not zero. Consequently, Quant is able to cope with just one signal per iTRAQ™ reporter ion. For the integration of peak areas, we introduced a minimum peak width, in order to provide this feature in that context. The sum of the signal intensities reflects the ion count recorded by the mass spectrometer more precisely than an integrated peak area, as shown in figure 3. Moreover, when summing up intensities the problem of just one reporter signal is not existent. The peaks are filtered by applying a threshold for the peak intensity. This is an option for the user, as the noise in mass spectra depends on the mass spectrometer that is used.

We improved the error estimation of other approaches [14] by adding precise error indication. Instead of taking only the maximum peak intensity as a basis of error estimation that has been formerly proposed [14], we use all peaks belonging to an iTRAQ™ reporter for precise error calculation. Additionally, we propagate the implications of the purity correction on the error estimation. When relative quantification is calculated, we propagate the estimated errors and use them for calculation of a quantification error. This is the maximum possible error and can be used as a quality indicator. If reporter peaks are missing for a label, the relative quantification cannot be performed. Thus, no zero values appear in the peptide ratio lists of the proteins and the log-transformation can be performed in all cases.

Multiple MS/MS spectra belonging to the same peptide sequence are not merged to one quantification value. We regard them as single measurements that are analyzed separately. Thus by using Quant, modified and unmodified peptides can be distinguished. Moreover, modified peptides might appear as outliers of the boxplot and can be analyzed separately. Some examples of this are included in the figures 5, 6, 7, 8, 9. If outliers are detected, the amino acid sequence should be analyzed in detail, and in some cases a new database search should be performed in order to confirm these sequences and to seek out further post-translational modifications, e.g. non iTRAQ™ labelled peptides because of N-terminal cyclation or acetylation of primary amino groups.

Quant uses MS/MS data extracted by other means and therefore is independent of any peak-picking method. Moreover it is independent of the mass spectrometer controlling software.

In comparison with Peakardt.FindPairs [31] that uses the mean value of peptide ratios for protein quantification, we use the median. This is statistically sound and correct, as peptide ratios are lognormal distributed (see figure 2) and therefore the mean value does not equal the median. Moreover, the median is robust against outliers that would have effects on the mean value. Therefore, there is no need to eliminate or to reject outliers. Moreover, Quant is able to point the user to outliers that should be analyzed further.

As a numeric tool for estimation of the quantification quality, we introduce the root-mean-square value (RMS) into protein quantification. This value is calculated from the relative errors of the peptide ratios. In contrast to the quality estimation by applying the standard deviation to the non-transformed data, the RMS is independent of the number of data points. Calculation of the standard deviation requires sufficient data points for doing a precise assumption on the underlying distribution of the data. Other tools, such as Peakardt.FindPairs [31], use the standard deviation *σ *of the non-transformed data as a quality measure. That approach uses *σ *and the Tschebyscheff-equation as basis for identification of outliers. This is needed for Peakardt.FindPairs, because the mean value is used as a parameter for protein quantification, which is sensitive to outliers. If the median would have been chosen, this problem would not occur.

Mascot™ 2.2 provides an analysis of iTRAQ™ data that is described online [32]. The lognormal distribution is employed. We could show that peptide ratios are from lognormal distribution and in consequence the use of the Shaprio-Wilk-test is the appropriate choice. We suggest not to rely on data with less than 5 observations when using this test, an upper limit for this procedure does not exist [33]. Mascot™ does not provide an experiment error or a bias within the result display. We could show that Mascot™ 2.2 bases on statistically correct and appropriate assumptions, concerning the iTRAQ™ evaluation.

ProteinPilot™ itself uses the same statistical approach as ProQuant™, but restricts peptides to unique ones. According to the information available with the trial version, the software estimates the experiment error (bias correction) with at least 20 protein ratios, although the median is applied. In contrast to Quant that makes use of the median and the LSE, ProteinPilot™ calculates the protein ratio by a weighted average. Similar to our approach, ProteinPilot™ yields a quality measure that is derived from the 95% confidence interval (error factor) which is calculated from the standard deviation in logspace.

In contrast to other quantification software that often are restricted to the use of only one protein identification algorithm, such as Mascot™ (Mascot™ 2.2), Sequest™ (RelEx – no iTRAQ™ capability), ProID™ (ProQuant™) or Paragon™ (ProteinPilot™), our method named Quant is independent of the identification algorithm. Moreover, Quant implements the purity correction including error propagation and precise error estimation. Additionally, we present reasons on an appropriate manner of intensity calculation as preprocessing for peptide ratio analysis.

The data presented in tables 3, 4 suggest that integration of non-unique peptides into calculation of protein quantification impairs the results in a negative way. The results generated by Quant are consistent with those produced by ProQuant™ as well as with Mascot™ 2.2. Because of the precise error propagation and the adequate visualization, the data obtained by using Quant is reliable.

## Conclusion

We have shown that relative quantification can be performed on data generated by tandem MS and iTRAQ™. We presented an analyzing method named Quant capable of calculating precise data, what has been shown by application to the protein standard mix supplied with the iTRAQ™ kit. The protein ratios of this standard have been calculated precisely from MS/MS spectra of the identification results.

We showed that restriction of the data evaluation to unique peptides is the only way of obtaining reliable quantification results at the protein level. Identification of unique peptides can be easily automated. Moreover, Quant is independent of the underlying protein identification software.

We have shown that a lognormal distribution fits the data of relative peptide quantification by applying the Shapiro-Wilk-test on the log-transformed data. Outliers can be identified by applying proper means of statistical tools, i.e. distribution analysis, boxplot, median, LSE and RMS. These are helpful as quality measures. We replaced peak area integration by sum of intensities, yielding reliable quantification results.

The methods presented here scale well with the protein and peptide ratios. The quality of the results yielded by Quant are not dependant of the peptide or protein ratios, but rather depend on the quality of the MS/MS experiment as well as on the protein identification and the MS/MS spectra, especially the scale of signal intensities is important. Therefore, and proven by the statistically sound system, the dynamic range of Quant is not limited by the inherent methods in comparison to the instrumental methods. Moreover, Quant provides a precise quality measure of the protein quantification by the RMS value.

The presented method is expandable to the 8-plex iTRAQ™ [34] as it is independent of the number of different labels.

Our data analysis method is more robust than other published software tools. Quant demonstrates improvements in peptide and protein quantification using iTRAQ™. Precise quantification data can be obtained when using error propagation and adequate visualization in conjunctions with consideration of an experiment error. Quant is shown to generate results that are consistent with those produced by ProQuant™ and Mascot™ 2.2, thus validating these systems.

## Availability and requirements

The MATLAB^® ^program scripts are freely available upon request from the authors and freely available via  and  under the GNU Lesser General Public License. A MATLAB^® ^installation is required for executing the scripts.

## List of abbreviations used

Å: Angström

ABI: Applied Biosystems/MDS-Sciex

ACN: acetonitrile

amu: atomic mass unit

BCA: bicinchoninic acid

Da: Dalton

DIGE: difference gel electrophoresis

DNA: desoxyribonuclein acid

DTA: file format for MS/MS spectrum data

EE: experiment error

EF: error factor of ProQuant™

FA: formic acid

ID: inner diameter

Id: database identifier of a protein

IQR: inter-quartile range

iTRAQ™: isobaric tag for relative and absolute quantitation

LSE: least squares estimator

μm: micrometre

mm: millimetre

mM: millimolar

MMTS: methyl methanethiosulfonate

MS: mass spectrometry

MS/MS: tandem mass spectrometry

NHS: N-hydroxy-succinimide

NN: No value available

pVal: p-value

RMS: root-mean-square value

SCX: strong cation exchange

## Authors' contributions

AB initiated the project and implemented the program in the laboratory. DA implemented the MATLAB^® ^scripts. MF and DA introduced precise error estimations and statistics to the project. AS and SP conducted the experiments and contributed with ideas and discussions. AB, SP and DA contributed equally to the manuscript. All authors have read and approved the final manuscript.

## Supplementary Material

Additional File 1 Archive containing the MATLAB^® ^scripts. This file contains the MATLAB^® ^scripts of Quant that can be executed with MATLAB^®^. Click here for file

Additional File 2 Documentation of the MATLAB^® ^scripts. This file contains the full documentation of the Quant scripts and explains the usage of the MATLAB^® ^scripts. Click here for file

Additional File 3 Archive containing the example data. This file contains the example data, that can be processed with the Quant scripts. Click here for file
