# Supplementary material for: Precise protein quantification based on peptide quantification using iTRAQ™
Source: BMC Bioinformatics. 2007 Jun 21;8:214. doi: 10.1186/1471-2105-8-214 (PMC1940031; doi:10.1186/1471-2105-8-214)
Supplement: Additional File 2 — Documentation of the MATLAB® scripts. This file contains the full documentation of the Quant scripts and explains the usage of the MATLAB® scripts. [file 1471-2105-8-214-S2.pdf]

Precise protein quantification based on peptide quantification using iTRAQ™

Andreas M. Boehm[1*], Stephanie Pütz[1], Daniela Altenhöfer[2], Albert Sickmann[1*], and Michael Falk[2*]

[1] Rudolf Virchow Center, DFG Research Center for Experimental Biomedicine, University of Wurzburg, (Protein Mass Spectrometry and Functional Proteomics), Wurzburg, D-97078, Germany
[2] Institute of Mathematics, University of Wuerzburg, Am Hubland, D-97074 Wuerzburg, Germany
[*] to whom correspondence should be addressed
Email: A. M. Boehm – ab@andiboehm.de, S. Pütz – stephanie.puetz@virchow.uni-wuerzburg.de, D. Altenhöfer – dani.altenhoefer@freenet.de, A. Sickmann - albert.sickmann@virchow.uni-wuerzburg.de, M. Falk – falk@mathematik.uni-wuerzburg.de

Documentation of the MATHLAB® scripts for iTRAQ™

This document describes the MATHLAB® Scripts designed for iTRAQ™ evaluation based on MS/MS.

Installation and running

An installation of MATHLAB® is required. Copy the files in one directory. Prepare the file `names01.txt` in an appropriate manner and run `startquantitraq` in order to start the peptide evaluation. Afterwards prepare the data files for the protein quantification as described below, create the list of result file names and start `startexperror`.

Precise relative peptide quantification

The MS/MS files of the iTRAQ™ experiment are analyzed and the relative peptide quantification is performed including a precise error estimation.

startquantitraq.m

Purpose: Start an iTRAQ™ peptide evaluation.
Process: The environment for an iTRAQ™ evaluation is prepared and some variables are set. Reads the text file `names01.txt` that lists one MS/MS spectrum per line and calls `quantitraq.m` once per spectrum. The spectra are expected in DTA format.
Input: The purity correction values of the iTRAQ™ kit are provided in the file `v.xls` as tab-separated values. The peak integration method is set by the variable `int` (0: sum of intensities, 1: trapezoid integration). The value of `a` determines the range for reporter ion search and the intensity error is provided by `e`. The minimal peak width for the case of just one present signal is defined by `width`.

Output: Peptide ratios per peptide for each reporter combination including the relative error estimation.

quantitraq.m

Purpose: Collect all reporter signals in an MS/MS spectrum and evaluate the data.
Process: The reporter signals are extracted from the MS/MS spectrum and the appropriate method of integration is applied (`sumquantitraq.m` or `flquantitraq.m`). The purity correction is performed by executing `pcquantitraq.m`. and the peptide ratios are calculated by `raquantitraq.m`.
Input: Selected integration method `int`, MS/MS spectrum `A`, range for reporter ion search `a`, purity correction values `v`, intensity error `e`, minimal peak dimension `width`, name of MS/MS spectrum `name`.
Output: A matrix containing the peptide ratios and a matrix with the corresponding relative errors. List of used peaks in the reporter area.

sumquantitraq.m

Purpose: Calculate the sum of intensities of reporter ion signals including an appropriate error estimation.
Process: Calculate the sum of intensities including an appropriate error estimation.
Input: Intensity error estimation `e` and the reporter signals `D`.
Output: Returns four reporter ion values including the corresponding error estimation.

flquantitraq.m

Purpose: Calculate the trapezoid integral of reporter ion signals including an appropriate error estimation.
Process: Calculate the sum of intensities including an appropriate error estimation.
Input: Intensity error estimation `e`, the reporter signals `D` and the minimal peak dimension applied in the case of just one reporter signal.
Output: Returns four reporter ion values including the corresponding error estimation.

pcquantitraq.m

Purpose: Correct the four reporter values and determine the appropriate relative errors.
Process: The correction of the four reporter values is performed and the corresponding relative errors are determined.
Input: Integration error estimation `e`, reporter values `A`, matrix `C` of purity correction values, name of MS/MS spectrum.
Output: Corrected reporter values including an error estimation. Matrix `C` of purity correction.

raquantitraq.m

Purpose: Calculate the peptide ratios from the corrected reporter values including the corresponding errors.
Process: The peptide ratios are calculated from the corrected reporter values. The corresponding errors are determined.
Input: Corrected reporter values including an error estimation.

Output: Peptide ratios per peptide for each reporter combination including relative error estimation.

Evaluation and correction of the experimental error

The MS/MS peptide identification must be performed externally by any protein identification software that generates a list of identifications. The peptide identification results must then be grouped and transformed in an appropriate manner preserving the linkage between the MS/MS data and the protein as well as the peptide sequence. The data files read in this procedure belong to a single experiment. They have five columns, whose first three contain information about the peptide and the spectrum, the forth represents the peptide ratio and the last column contains the relative error.

startexperror.m

Purpose: Start an iTRAQ™ protein evaluation.
Process: Reads the text file `names01.txt` that lists one iTRAQ™ peptide result per line and calls `experror.m` and `ratiosexperror` once per entry. The results are stored in files.
Input: Read from the file `names01.txt`. Data files: column 4 contains the peptide ratios, column 5 the corresponding error.
Output: Stored in files with generated file names.

experror.m

Purpose: Performs the analysis and calculation of the experimental error.
Process: Zero peptide ratios are removed from the input as they result from missing reporter ion values and therefore have to be ignored. This is done by `killzero.m`. Calculation of the median of the data as experimental error and the appropriate relative error. The data is then log-transformed by executing `logtrans.m`. A normal-probability-plot is generated and stored by calling `qplot.m`.
Input: Peptide quantification data `data`, index number `k` and the filename `name`.
Output: Experimental error and appropriate relative error

killzero.m

Purpose: Removes zero entries from the input data.
Process: Zero peptide ratios are removed from the input.
Input: Data matrix `data`.
Output: Data matrix (two columns) without zero values.

logtrans.m

Purpose: Performs the log-transformation.
Process: The log-transformation is done.
Input: Data matrix `data`.
Output: Data matrix with log-transformed columns 1 and 2.

qplot.m

Purpose: A normal-probability-plot is generated and saved in a file.
Process: The normal-probability-plot of the input data is created and saved. The mean value and standard deviation is calculated.

Input: Data matrix `data` with log-transformed data.
Output: Normal-probability-plot stored in a graphic file.

ratiosexperror.m

Purpose: Application of the experimental error.
Process: Scales ratios and corresponding errors.
Input: Ratio with relative errors `D`, Filename `name`, experimental error `expf`.
Output: Shifted ratios (column 1) and errors (column2), also stored in files with generated file names.

Precise relative protein quantification and plotting the protein quantification

The data files read in this procedure belong to a single protein each. They have two columns. The first represents the peptide ratio and the second column contains the relative error. The data has to be sorted by the proteins the peptides belong to. A list named `block.xls` contains the proteins in the first column and the number of peptides in the second column.

startplotitraq.m

Purpose: Calculates the quantification and quality values. Plots the iTRAQ™ evaluation data. Processes list of result files `names02.txt`.
Process: Calls `plotitraq.m` once per protein data set.
Input: Data containing peptide values and relative errors, block listing the proteins with number of corresponding peptides.
Output: List of quantification and quality values for each protein, one line per protein in this order: (k (number of peptides), median, IQR, RMS, LSE, mean, standard deviation)

plotitraq.m

Purpose: Calculates the Quantification and Quality values and plots the iTRAQ™ evaluation data for each protein.
Process: Calculates the quantification of the protein by the peptide values, evaluates the quality values, creates plots.
Input: Ratios, errors, number of peptides for this protein
Output: Quantification and quality values for this protein, boxplot and error plot stored in files.
